# The Correlation Between Cracked Teeth and National Insurance Coverage of Dental Implants in South Korea: A Retrospective Cohort Analysis

**DOI:** 10.3390/jcm14155507

**Published:** 2025-08-05

**Authors:** Se Hoon Kahm, YoungHa Shim, SungEun Yang

**Affiliations:** 1 Department of Dentistry, Eunpyeong St. Mary’s Hospital, College of Medicine, The Catholic University of Korea, Seoul 03312, Republic of Korea; implant@catholic.ac.kr; 2 Department of Conservative Dentistry, Seoul St. Mary’s Dental Hospital, College of Medicine, The Catholic University of Korea, Seoul 06591, Republic of Korea

**Keywords:** cracked teeth, dental implant, National Health Insurance, occlusion, clinical data warehouse, retrospective study

## Abstract

**Background/Objectives**: The expansion of National Health Insurance (NHI) coverage for dental implants in South Korea has substantially increased implant placements among older adults. While implants offer functional and esthetic benefits, their lack of periodontal ligaments alters occlusal force distribution, potentially increasing biomechanical stress on adjacent or opposing teeth. This study aimed to investigate the association between the increased number of dental implants and the incidence of cracked teeth following the introduction of implant insurance. **Methods**: A retrospective analysis was conducted using the Clinical Data Warehouse of Seoul St. Mary’s Dental Hospital. Patients who underwent molar crown restorations between 2014 and 2022 were included. The incidence and clinical features of cracked teeth were compared before (2014–2015) and after (2016–2022) the introduction of implant insurance. Statistical analyses assessed differences in symptom presentation, pulp status, and treatment outcomes. **Results**: Among 5044 molars restored with crowns, 1692 were diagnosed with cracks. The incidence of cracked teeth significantly increased after NHI coverage for implants (25.5% vs. 32.6%, *p* < 0.001). Cases after insurance implementation showed fewer signs and symptoms at initial presentation (67.4% vs. 50.0%, *p* < 0.001), reduced irreversible pulpitis (37.2% vs. 25.8%, *p* < 0.001), and increased preservation of pulp vitality (46.9% vs. 57.8%, *p* < 0.001). These shifts may reflect changes in occlusal adjustment practices and earlier clinical intervention. **Conclusions**: The findings suggest a temporal link between increased implant placement and the rising incidence of cracked teeth. Implant-induced occlusal changes may contribute to this trend. Careful occlusal evaluation and follow-up are essential after implant placement, and further prospective studies are warranted to confirm causality and refine prevention strategies.

## 1. Introduction

Dental implants have become increasingly popular as a replacement for missing teeth, primarily because they preserve adjacent teeth and achieve high esthetic success. Unlike natural teeth, which are connected to the alveolar bone via periodontal ligaments, implants are directly osseointegrated into the bone. Periodontal ligaments not only allow physiological mobility but also disperse stress, withstand occlusal forces, and function as mechanoreceptors, playing a vital role in regulating excessive occlusal forces [1]. However, implants do not have this regulatory mechanism, which makes them more vulnerable to excessive occlusal forces [1,2]. Furthermore, adjusting occlusion in implant restorations to prevent overloading may increase stress on the surrounding natural teeth. It is hypothesized that implants, when placed next to physiologically mobile natural teeth, may lead to an increase in cracks or fractures in the neighboring teeth due to the loosening of the proximal contact area.

The exact occlusal relationship between implants and adjacent natural teeth has not yet been fully clarified [3,4,5,6,7,8]. However, a 2016 study by Rosen et al. suggested a potential link between the presence of implants and the occurrence of vertical root fractures in adjacent endodontically treated teeth [1]. This study found that reducing the load on implant prostheses results in an increased load on adjacent teeth, which may lead to vertical root fractures. Additionally, excessive occlusal forces on teeth next to implants, coupled with diminished proprioception and reduced fracture resistance in endodontically treated teeth, could also contribute to vertical root fractures. In a subsequent 2017 study, Rosen et al. observed cracked teeth in non-endodontically treated teeth adjacent to implants more than a year after the implants were placed [9]. Therefore, it is important to further investigate the correlation between implant placement and the occurrence of cracked teeth in surrounding teeth.

Countries differ significantly in public dental insurance for implants. For example, Sweden and Germany offer some level of public support for dental care, while countries like the USA, Spain, and Switzerland provide little or no public insurance for implants in Table 1 [10]. Studies show that older adults in countries with comprehensive public dental coverage tend to have better oral health outcomes and more equitable access to dental care compared to those in countries without such coverage [10]. In South Korea, National Health Insurance (NHI) began covering dental implants for older adults on 1 July 2014. Initially, this coverage was available to partially edentulous patients aged 75 or older, supporting up to two implants. The eligibility criteria expanded over time, including patients aged 70 or older from 1 July 2015, and those aged 65 or older from 1 July 2016. Furthermore, on 1 July 2018, the patient’s cost-sharing requirement decreased from 50% to 30%. Consequently, there was a notable increase in the number of patients receiving implants, rising from 398,320 in 2016 to 574,100 in 2017, and reaching 582,837 in 2018. The data also show a significant surge in implant placements, with a 44% increase between 2016 and 2017, amounting to approximately 170,000 additional cases.

Based on the rapid increase in implant placements, the study divided the timeframe into two phases—2014–2015 and 2016–2022—to compare and analyze the incidence of cracked teeth associated with implant placement. Additionally, it aimed to investigate the correlation between the rising number of implant placements and the incidence of cracked teeth.

## 2. Materials and Methods

### 2.1. Ethical Considerations

This study was conducted with the approval of the Institutional Review Board (IRB) of Seoul St. Mary’s Hospital, The Catholic University of Korea (Approval Number: KC22WISB0849). Access to electronic medical records and radiographic data through the Clinical Data Warehouse (CDW) was granted by the Institutional Data Access Committee (Approval Number: 230713002).

### 2.2. Data Processing

Data for patients who visited the dental clinics of Seoul St. Mary’s Hospital between 2014 and 2022 were extracted from the Catholic Medical Center nU CDW, Catholic University of Korea, Seoul, Korea. All used data was encoded and anonymized through the extraction system of CDW from electronic medical record (EMR) data. This retrospective cohort study was based on electronic medical records from a single tertiary care hospital. All data were de-identified using encoding protocols to ensure that individual patients could not be identified. As the dataset contained no personally identifiable information, the study posed no risk of physical or psychological harm to participants. The CDW system utilized in this study has been collecting clinical data since the hospital’s inception since 1997. This includes prescriptions, pathology results, radiographs, and other patient information. The data is anonymized, facilitating the rapid gathering and analysis of correlations among various data points. The anonymization of patient data was achieved by assigning random numbers through the CDW system. Additionally, panoramic X-ray information was analyzed using the Enterprise Data Platform (EDP) system, ensuring that patient privacy was maintained.

### 2.3. Case Selection

The study focused on patients who underwent crown treatments at the Department of Conservative Dentistry at Seoul St. Mary’s Hospital from 2014 to 2022. Patients who received crown restorations were identified using specific treatment codes.

Exclusion criteria. Cases involving anterior teeth or trauma in patients under 19 years of age were excluded. Cases involving previous trauma were excluded due to their increased susceptibility to additional cracks or root fractures, which could confound the analysis of factors specific to age-related or occlusal-stress-induced crack development in adults. Similarly, patients under 19 years of age were excluded because this demographic often presents with mixed dentition and incompletely established occlusion, making their fracture patterns and long-term prognosis distinct from those in a mature adult dentition. Additionally, anterior teeth were excluded as their occlusal forces and relationship with opposing dentition, influenced by factors such as angulation, can lead to distinct patterns of inappropriate occlusal stress, which would complicate the analysis focused on the posterior dentition’s response to implant placement.

Inclusion criteria. Cracked teeth were identified based on diagnostic codes S0252, S0253, S0254, S0255, S0256, and S0259, according to the Korean Standard Classification of Diseases (KCD), which is based on the International Statistical Classification of Diseases and Related Health Problems, 10th Revision (ICD-10). A total of 5044 teeth that had undergone crown restoration were included in the analysis, among which 1692 were diagnosed with cracks. The relationship with implants was assessed using implant prescription codes (Insurance: CUB127; Non-insurance: CDT0260-3).

### 2.4. Collected Data

For teeth diagnosed with cracks, the analysis included several preoperative factors: patient age and gender, presence and nature of pain, masticatory discomfort, intraoral location, number of fracture lines, dental caries, extent of periodontal tissue damage, pulp status, and presence of implants prior to restoration. Post-treatment factors analyzed encompassed pain reduction, condition of periodontal tissues, and prognosis.

The extent of periodontal tissue damage was categorized based on the depth of the periodontal pockets: less than 3 mm, more than 4 mm but less than 6 mm, and more than 6 mm. The status of the pulp was classified as normal pulp, reversible pulpitis, irreversible pulpitis, or pulpal necrosis. The collected variables are summarized in Table 2.

### 2.5. Statistical Analysis

The study divided the analysis period into three phases—2014–2015, 2016–2019 and 2020–2022—due to a sharp rise in the number of implant placements observed among 2015–2016 and 2019–2020. The various manifestations of cracked teeth associated with implant placement were examined across these time frames. To assess significant temporal changes, yearly comparisons of cracked teeth incidence rates were performed using the chi-squared test, with the study period’s average rate used as the expected value.

Differences between various observed variables, such as sex, tooth location, and degree of symptom improvement, in relation to the presence or absence of implants were evaluated. The hypothesis posited that there would be no significant differences. The Fisher exact tests and Mantel-Haenszel chi-square tests were used for this evaluation.

All statistical analyses were conducted using R language version 4.2.2 (R Foundation for Statistical Computing, Vienna, Austria) and the TnF program version 4.0 (YooJin BioSoft, Goyang-si, Korea). Data were presented as sample counts and percentages (N (%)). We compared the proportion of cracked teeth among patients who received crown restorations before and after the introduction of implant insurance using chi-squared tests. The Bonferroni method was applied to correct for multiple tests. Associations between various risk factors before and after the introduction of implant insurance were analyzed using the chi-square or Fisher exact test.

## 3. Results

### 3.1. Trends in Cracked Teeth Incidence by Year

**Figure 1:**
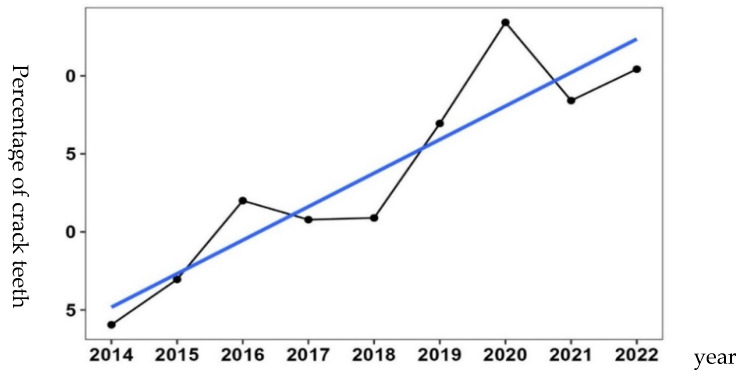
Trends in Cracked Teeth Incidence by Year. Increase in cracked teeth following implant insurance expansion. The blue line indicates the trend line showing the annual increase.

In patients who underwent crown restoration, there was a significant increase in the incidence rate of cracked teeth over the years (*p* < 0.001, Table 3, Figure 1).

When comparing the incidence rate of cracked teeth before and after NHI coverage of dental implants for older adults, the rate was 25.5% (288 out of 1128 patients) before NHI coverage and 35.9% (1404 out of 3916 patients) after NHI coverage. The two-sample proportion test revealed a statistically significant difference between the two periods (*p* < 0.001) (Table 4).

### 3.2. Analysis of Correlations Between Risk Factors and NHI Coverage of Dental Implants for Older Adults

The correlation between risk factors for cracked teeth and the introduction of implant insurance, comparing the periods 2014–2015 (before insurance), 2016–2019 and 2020–2022 (after insurance I, II), is shown in Table 5. The proportion of patients aged 70 and older with cracked teeth increased from 11.1% before NHI coverage of dental implants for older adults to 15.6% and 18.9% after NHI coverage. The symptoms and signs of cracked teeth were present in 67.4% (194 out of 288 patients) before NHI coverage but decreased to 50.3% (397 out of 789 patients) and 49.7% (297 out of 598 patients) after NHI coverage, indicating an increase in the proportion of asymptomatic cases. The incidence of biting pain also decreased from 51.9% (150 out of 289 patients) before NHI coverage to 30.6% (353 out of 791 patients) and 33.8% (204 out of 603 patients) after NHI coverage.

Regarding pulp status, the proportion of cases with normal pulp increased from 46.9% before NHI coverage to 57.2% and 58.5% after NHI coverage. Concurrently, the incidence of irreversible pulpitis or pulp necrosis decreased from 37.2% to 28.3% and 22.6%. These findings indicate an increase in the proportion of cases with normal pulp and a reduction in the proportion of cases with irreversible pulpitis or pulp necrosis.

The treatment methods for cracked teeth have evolved following the NHI’s extension of dental implant coverage to older adults. Prior to this insurance coverage, 24.7% of patients opted solely for crown restoration, whereas 75.3% underwent root canal treatment before receiving a crown restoration. After the introduction of NHI coverage for dental implants in older adults, the percentage of patients choosing only crown restoration rose to 41.1% and 64.9%, and those undergoing root canal treatment decreased to 58.9% and 34.6%. Additionally, the proportion of patients receiving root canal treatment followed by crown restoration significantly dropped from 74.0% before NHI coverage to 55.1% and 32.3% afterward. However, there was no significant change in the proportion of patients who received root canal treatment after crown restoration.

Other factors, such as sex, the presence of caries, gingival swelling, the type of previous restorations, cervical abrasion, the presence of periapical lesions, and tooth location, did not show significant correlations.

## 4. Discussion

### 4.1. Evolving Treatment Paradigms and Diagnostic Challenges in Cracked Teeth

In this study, we observed a notable trend toward earlier implant placement following the extraction of cracked teeth, especially after 2016. This shift suggests an evolving treatment paradigm in which clinicians may increasingly favor immediate or early-stage replacement with implants rather than attempting prolonged tooth preservation through endodontic or restorative approaches. This may reflect the growing preference for more predictable long-term solutions, as well as systemic influences on treatment decisions.

Diagnosing cracked teeth remains one of the most challenging aspects in clinical dentistry due to the variable and often nonspecific nature of symptoms. These may include thermal sensitivity, pain upon mastication, or localized periodontal pocketing, but such findings are neither consistent nor pathognomonic [11,12]. Radiographic imaging often fails to reveal the presence or extent of the crack, and even with magnification, definitive identification may be delayed until restorative failure or irreversible pulpitis occurs [13,14].

This diagnostic ambiguity often results in treatment delays or empirical interventions, such as occlusal adjustment or provisional restorations, until the prognosis becomes clearly unfavorable. Our data support this pattern, showing that many teeth were extracted and replaced with implants within 12 months of diagnosis—suggesting that in many cases, preservation was either deemed unfeasible or was attempted but ultimately failed.

### 4.2. Influence of Health Policy and Systemic Factors on Treatment Decisions

The observed increase in implant placement after 2016 appears to correspond temporally with the staged expansion of Korea’s National Health Insurance coverage for dental implants, which included those aged ≥75 years in 2014 and was expanded to patients aged ≥65 in 2016 [15]. While this policy has significantly enhanced treatment accessibility for older adults, it may have unintentionally influenced clinical decision-making by lowering economic barriers to implant placement. In particular, patients with borderline or uncertain tooth prognosis—such as those with cracked teeth—may have become more likely to choose extraction and implant placement as a definitive, covered solution.

The influence of public health insurance on treatment patterns has been previously documented in dental literature. According to Kim et al. [16], the implementation of implant insurance in Korea led to a marked increase in implant surgeries, especially among patients with posterior tooth loss. This was not solely due to increased demand, but also reflected clinicians’ evolving attitudes toward implant treatment as a first-line solution under the insurance framework. Similarly, Lee and colleagues [17] found that insurance coverage substantially impacted patients’ treatment preferences, particularly in cases of questionable prognosis.

Another potential factor is the increasing predictability and success of modern implant placement. Numerous studies have reported high long-term survival rates and improved prosthetic outcomes with implant-supported restorations, even in elderly or medically compromised populations [18]. In contrast, the outcomes of cracked teeth are variable, with success rates depending heavily on the extent of the crack, occlusal forces, and restorability [19,20]. Clinicians may therefore perceive implants as a more favorable option in terms of longevity, patient satisfaction, and procedural simplicity. It is also important to recognize the role of patient preference in this shift. As treatment information becomes more accessible through online platforms and social media, patients are increasingly involved in shared decision-making. When faced with uncertain outcomes and the possibility of retreatment, many may opt for what they perceive as a more definitive and technologically advanced solution—i.e., an implant [21].

### 4.3. Biomechanical Considerations: Implant Occlusion and Adjacent Tooth Health

Recent literature consistently sheds light on the intricate relationship between dental implants and complications in adjacent natural dentition, thereby strengthening the context of our findings. A retrospective cohort study by Chen et al., subsequently commented upon by Afrashtehfar et al., unequivocally demonstrated a significantly elevated risk of tooth loss in teeth adjacent to dental implants compared to non-adjacent teeth, with root fracture identified as the primary etiology [22,23]. This highlights the potential for implants to act as an iatrogenic factor contributing to complications in neighboring teeth, underscoring the critical need for meticulous planning and protective measures during implant placement. Conversely, a systematic review by Di Fiore et al. explored the potential association between occlusal overload and peri-implant bone loss, although it emphasized methodological limitations and the scarcity of quantifiable assessment methods in existing research, calling for further investigation with advanced digital tools [24]. Concurrently, Sheridan et al. provided a comprehensive review on the distinct biomechanical responses of natural teeth versus implants to occlusal forces, asserting that implant overload can lead to biomechanical failures and marginal bone loss, and thus necessitating precise occlusal management guidelines [25]. Collectively, these contemporary reviews and studies reinforce our hypothesis regarding the impact of implant placement on adjacent tooth health, particularly concerning fracture incidence, and underscore the imperative for clinicians to integrate comprehensive occlusal evaluation and tailored follow-up care into implant treatment protocols.

Biomechanical factors, including occlusal design and prosthetic materials, may contribute to cracked teeth development. The concept of implant-protective occlusion aims to minimize the occlusal load on implants by reducing lateral forces and the contact area [1,11,12,15]. However, this approach may shift the functional load to adjacent natural teeth, potentially increasing the crack risk [1,3,4,8,11,13,19]. One study reported that 78% of patients with cracked teeth had multiple implants [9], supporting this hypothesis. In our study, women showed a slightly higher prevalence of cracked teeth (52.4%), consistent with some prior findings [9], though others report a male predominance [14]. Cracks were most common in patients in their 50s and 60s, possibly reflecting age-related reductions in dentin resilience [14,21]. Over half of the affected teeth had prior restorations, predominantly metal-based, aligning with previous associations between amalgam and crack incidence [9]. Periodontal pocket depth >6 mm is a known negative prognostic factor [14,16], though most cases in our cohort had shallow pockets (<3 mm), which slightly increased in frequency post-insurance expansion—possibly indicating earlier diagnosis [26]. Prosthetic material, such as zirconia, may also increase wear on opposing dentition, highlighting the need for careful material selection [18,20]. Further studies are needed to clarify how occlusion and prosthetic factors contribute to crack progression.

### 4.4. Global Trends and Stress-Related Dental Issues, Including COVID-19 Impact

In this study, we observed a marked increase in the incidence of cracked teeth following the introduction of insurance-covered dental implants in Korea, with a further significant rise during the COVID-19 pandemic. This pattern was not limited to Korea but is consistent with global trends. For example, a Romanian study reported that the incidence of cracked teeth and vertical root fractures significantly increased during the COVID-19 pandemic, with most cases occurring in root canal-treated teeth and often requiring extraction, likely due to heightened stress and reduced dental visits during this period [27]. To further support this trend, studies from both the United States and the United Kingdom have reported similar increases in the incidence of cracked teeth during the COVID-19 pandemic [28,29]. For instance, an infodemiological analysis found a significant rise in the burden of cracked teeth in the US, likely linked to pandemic-related stress and behavioral changes. Additionally, research in Saudi Arabia has discussed the impact of the pandemic on oral health behaviors and the increased demand for emergency dental treatments, suggesting a broader pattern of heightened dental issues, including tooth fractures, during this period [30]. Given these findings, it is important to develop detailed guidelines for dental care and precise occlusal adjustment during periods of heightened stress, such as a pandemic. Enhanced preventive strategies and individualized management of restorations will be essential to mitigate the increased risk of cracked teeth observed under such stressful conditions [31].

### 4.5. Limitations and Future Directions

Nevertheless, this study has several limitations. The retrospective design restricts access to nuanced clinical data, including exact diagnostic methods, crack depth or direction, and patient-reported symptoms. Furthermore, we could not evaluate other potentially influential factors such as detailed occlusal status, practitioner variation, systemic health, or precise crack characteristics. The lack of standardization in treatment protocols further complicates interpretation, particularly regarding standardized occlusal assessments post-implant placement. Despite these limitations, our findings highlight a clinically and socially relevant trend: the growing influence of health policy, technology, and social behavior on dental treatment decisions. The management of cracked teeth represents a unique intersection of diagnostic uncertainty, patient psychology, and systemic factors. Moving forward, a multicenter prospective study will be essential to confirm causality and standardize occlusal evaluations post-implant placement, in addition to incorporating standardized diagnostic criteria, clinical outcomes, patient preferences, and policy-level analysis. Specifically, more detailed research is needed on factors that directly influence crack development, such as the positional relationship between implants and cracked teeth (e.g., adjacent teeth, opposing teeth). These insights underscore the importance of individualized treatment planning and careful occlusal management following implant therapy.

## 5. Conclusions

The study reveals a notable increase in the incidence of cracked teeth following the introduction of dental implant insurance coverage in South Korea. This trend seemed to correlate with the rapid escalation in the number of implant placements, suggesting a possible link between the implant procedures and the emergence of cracks in adjacent or opposing natural teeth. Importantly, the nature of cracked teeth cases has changed, showing more asymptomatic presentations and fewer cases requiring root canal treatment. This change could be due to earlier detection and changes in occlusal dynamics brought about by implant prostheses. These observations highlight the critical need for thorough occlusal evaluation and precise adjustment after implant placement, as well as consistent follow-up care to identify and address potential complications. Continued longitudinal research is crucial to establish a direct causal relationship between implant placement and cracked teeth and to clarify the complex interplay of risk factors contributing to this phenomenon.

## Figures and Tables

**Table 1:** Comparison of Public Dental Implant and Prosthodontic Coverage for Older Adults.

Country	Public Coverage for Older Adults	Key Aspects of Dental and Prosthodontic Coverage
South Korea	Yes	Publicly covered for seniors aged 65+. Up to two implants per lifetime covered with 30% co-payment.
Sweden	Yes	Fixed public subsidies for all general dental treatment, including prosthodontics. Additional support for high-cost cases.
Germany	Yes	Wide public coverage for basic/conservative treatments. Partial coverage for prosthodontics (50–60%).
USA	No	Primarily privately funded; only 5% publicly funded.
Spain	No	No public funding for dental care, except extractions.
Switzerland	No	No general public coverage; exceptions for severe diseases. Low private coverage uptake.

**Table 2:** Variables.

Variable	Subgroup
Sex	F, M
Age group	20–29, 30–39, 40–49, 50–59, 60–69, ≥70
Sign and Symptoms	Absent, Present
Biting pain	Absent, Present
Pulpitis	Absent, Present
Pulp status	Normal, Reversible pulpitis, Irreversible pulpitis + Pulp necrosis + Pulpless
Gingival swelling	Absent, Present
Apical lesion	Absent, Present
Pocket depth	<3 mm, 4–6 mm, >6 mm
Treatment	endodontic tx. + crown, resin + crown, resin + crown → endodontic tx.
Procedure	endodontic tx. + crown, resin + crown, resin + crown → endodontic tx.
Location of teeth	Mandibular first molar, Mandibular first premolar, Mandibular second molar, Mandibular second premolar,
	Maxillary first molar, Maxillary first premolar, Maxillary second molar, Maxillary second premolar

**Table 3:** Yearly Cracked Teeth Incidence Rates.

Year	Total Crown No.	Total Crack No.	Crack%
2014	553	133	24.1
2015	575	155	27.0
2016	550	176	32.0
2017	588	181	30.8
2018	670	207	30.9
2019	609	225	36.9
2020	631	274	43.4
2021	492	189	38.4
2022	376	152	40.4
*p* value			<0.001
Adj. *p* value			<0.001
Reg. coef. (95% CI)			2.148 (1.449–2.848)
Reg. coef. *p* value			<0.001

**Table 4:** Comparison of cracked teeth before and after implant insurance.

Period	Total	Crack	No Crack	*p* Value
Before insurance	1128 (22.4)	288 (25.5)	840 (74.5)	<0.001
After insurance	3916 (77.6)	1404 (35.9)	2512 (64.1)	
Total	5044 (100.0)	1692 (33.5)	3352 (66.5)	
*p* value		<0.001		

**Table 5:** Correlations between risk factors and National Health Insurance (NHI) coverage of dental implants for older adults.

Variable	Subgroup	Before Insurance 2014–2015	After Insurance I 2016–2019	After Insurance II 2020–2022	Total	*p* Value	*p* Value for Proportion Test
N(%)		289 (17.2)	791 (47.0)	603 (35.8)	1683		
Sex	Total	288 (17.2)	789 (47.1)	598 (35.7)	1675 (100.0)	0.286	
	F	146 (50.7)	418 (53.0)	335 (56.0)	899 (53.7)		0.286
	M	142 (49.3)	371 (47.0)	263 (44.0)	776 (46.3)		0.286
Age group	Total	288 (17.2)	789 (47.1)	598 (35.7)	1675 (100.0)	0.017	
	20–29	7 (2.4)	19 (2.4)	16 (2.7)	42 (2.5)		0.947
	30–39	44 (15.3)	80 (10.1)	56 (9.4)	180 (10.7)		0.022
	40–49	55 (19.1)	142 (18.0)	82 (13.7)	279 (16.7)		0.050
	50–59	75 (26.0)	215 (27.2)	152 (25.4)	442 (26.4)		0.737
	60–69	75 (26.0)	210 (26.6)	179 (29.9)	464 (27.7)		0.309
	≥70	32 (11.1)	123 (15.6)	113 (18.9)	268 (16.0)		0.011
Sign and Symptoms	Total	288 (17.2)	789 (47.1)	598 (35.7)	1675 (100.0)	<0.001	
	Absent	94 (32.6)	392 (49.7)	301 (50.3)	787 (47.0)		<0.001
	Present	194 (67.4)	397 (50.3)	297 (49.7)	888 (53.0)		<0.001
Biting pain	Total	289 (17.2)	791 (47.0)	603 (35.8)	1683 (100.0)	<0.001	
	Absent	139 (48.1)	549 (69.4)	399 (66.2)	1087 (64.6)		<0.001
	Present	150 (51.9)	242 (30.6)	204 (33.8)	596 (35.4)		<0.001
Old restoration	Total	289 (17.2)	791 (47.0)	602 (35.8)	1682 (100.0)	0.076	
	Absent	134 (46.4)	390 (49.3)	260 (43.2)	784 (46.6)		0.076
	Present	155 (53.6)	401 (50.7)	342 (56.8)	898 (53.4)		0.076
Cervical abrasion	Total	289 (17.2)	791 (47.0)	603 (35.8)	1683 (100.0)	0.015	
	Absent	263 (91.0)	729 (92.2)	528 (87.6)	1520 (90.3)		0.015
	Present	26 (9.0)	62 (7.8)	75 (12.4)	163 (9.7)		0.015
Pulpitis	Total	289 (17.2)	791 (47.0)	603 (35.8)	1683 (100.0)	0.782	
	Absent	209 (72.3)	584 (73.8)	436 (72.3)	1229 (73.0)		0.782
	Present	80 (27.7)	207 (26.2)	167 (27.7)	454 (27.0)		0.782
Pulp status	Total	288 (17.2)	789 (47.1)	598 (35.7)	1675 (100.0)	<0.001	
	Normal	135 (46.9)	451 (57.2)	350 (58.5)	936 (55.9)		0.003
	Reversible pulpitis	46 (16.0)	115 (14.6)	113 (18.9)	274 (16.4)		0.096
	Irreversible pulpitis + Pulp necrosis + Pulpless	107 (37.2)	223 (28.3)	135 (22.6)	465 (27.8)		<0.001
Gingival swelling	Total	288 (17.2)	789 (47.1)	598 (35.7)	1675 (100.0)	0.599	
	Absent	274 (95.1)	761 (96.5)	573 (95.8)	1608 (96.0)		0.599
	Present	14 (4.9)	28 (3.5)	25 (4.2)	67 (4.0)		0.599
Apical lesion	Total	288 (17.2)	789 (47.1)	598 (35.7)	1675 (100.0)	0.144	
	Absent	256 (88.9)	682 (86.4)	537 (89.8)	1475 (88.1)		0.144
	Present	32 (11.1)	107 (13.6)	61 (10.2)	200 (11.9)		0.144
PD	Total	289 (17.2)	791 (47.0)	602 (35.8)	1682 (100.0)	0.265	
	<3 mm	206 (71.3)	615 (77.7)	462 (76.7)	1283 (76.3)		0.082
	4–6 mm	70 (24.2)	147 (18.6)	115 (19.1)	332 (19.7)		0.106
	>6 mm	13 (4.5)	29 (3.7)	25 (4.2)	67 (4.0)		0.797
Treatment	Total	288 (17.2)	789 (47.1)	598 (35.7)	1675 (100.0)	<0.001	
	endo + crown	217 (75.3)	465 (58.9)	207 (34.6)	889 (53.1)		<0.001
	resin + crown	71 (24.7)	324 (41.1)	390 (65.2)	785 (46.9)		<0.001
	resin + crown → endo	0 (0.0)	0 (0.0)	1 (0.2)	1 (0.1)		0.406
Procedure	Total	288 (17.2)	789 (47.1)	598 (35.7)	1675 (100.0)	<0.001	
	endo + crown	213 (74.0)	435 (55.1)	193 (32.3)	841 (50.2)		<0.001
	resin + crown	71 (24.7)	324 (41.1)	388 (64.9)	783 (46.7)		<0.001
	resin + crown → endo	4 (1.4)	30 (3.8)	17 (2.8)	51 (3.0)		0.117
Location of teeth	Total	289 (17.2)	790 (47.1)	598 (35.7)	1677 (100.0)	0.871	
	Mandibular first molar	53 (18.3)	184 (23.3)	147 (24.6)	384 (22.9)		0.109
	Mandibular first premolar	3 (1.0)	9 (1.1)	6 (1.0)	18 (1.1)		0.969
	Mandibular second molar	71 (24.6)	187 (23.7)	123 (20.6)	381 (22.7)		0.280
	Mandibular second premolar	10 (3.5)	22 (2.8)	15 (2.5)	47 (2.8)		0.723
	Maxillary first molar	74 (25.6)	173 (21.9)	137 (22.9)	384 (22.9)		0.439
	Maxillary first premolar	12 (4.2)	39 (4.9)	30 (5.0)	81 (4.8)		0.838
	Maxillary second molar	44 (15.2)	125 (15.8)	99 (16.6)	268 (16.0)		0.867
	Maxillary second premolar	22 (7.6)	51 (6.5)	41 (6.9)	114 (6.8)		0.798
Caries	Total	288 (17.2)	788 (47.1)	598 (35.7)	1674 (100.0)	0.015	
	Absent	211 (73.3)	603 (76.5)	416 (69.6)	1230 (73.5)		0.015
	Present	77 (26.7)	185 (23.5)	182 (30.4)	444 (26.5)		0.015

Patient number (%) are computed in each subgroup. *p* value: *p* value computed using chi-squared test or Fisher’s exact test to analyze significance of association between symptom and each risk factor. *p* value for proportion test: *p* value computed using two sample proportion test to analyze significance of difference between ratios of patients with symptom “Absent” and “Present”.

## Data Availability

Data can only be provided or accessed from the corresponding author upon reasonable request.

## Abbreviations

The following abbreviations are used in this manuscript:

NHI	National Health Insurance
CDW	Clinical Data Warehouse
EDP	Enterprise Data Platform
ICD	International Statistical Classification of Diseases and Related Health Problems
KCD	Korean Standard Classification of Diseases
